# Recurrent Posterior Reversible Encephalopathy Syndrome Potentially Related to AIDS and End-Stage Renal Disease: A Case Report and Review of the Literature

**DOI:** 10.1155/2012/914035

**Published:** 2012-10-10

**Authors:** Olivia Hui-Chiun Chang, Alexandra Stanculescu, Chi Dola, William Benjamin Rothwell

**Affiliations:** ^1^Department of Internal Medicine, Tulane University School of Medicine, SL-12, 1430 Tulane Avenue, New Orleans, LA 70112-2699, USA; ^2^Department of Obstetrics and Gynecology, Tulane University School of Medicine, SL-12, 1430 Tulane Avenue, New Orleans, LA 70112-2699, USA

## Abstract

Posterior reversible encephalopathy syndrome (PRES) is a clinicoradiological syndrome that is characterized by clinical features including headache, altered mental status, cortical blindness, seizures, and other focal neurological signs as well as subcortical edema without infarction on neuroimaging. Under the umbrella of hypertensive encephalopathy, PRES is defined by reversible cerebral edema due to dysfunction of the cerebrovascular blood-brain barrier unit. The pathophysiology of PRES is thought to result from abnormalities in the transmembrane flow of intravascular fluid and proteins caused by two phenomena: one, cerebral autoregulatory failure and two, loss of integrity of the blood-brain barrier. PRES is not a common disease in patients with human immunodeficiency virus (HIV) and AIDS with only three previously reported cases. Both the HIV and end-stage renal disease appear to further compromise the blood brain barrier. Although uncommon, PRES recurrence has been described. To the best of our knowledge, this is the first report demonstrating recurrent PRES in a HIV patient on hemodialysis for end-stage renal disease.

## 1. Case Report

A 28-year-old woman with a history of human immunodeficiency virus (HIV), CD4 count of 28, HIV nephropathy on hemodialysis, and hypertension presented with intractable nausea and vomiting. She was known to be poorly compliant with medications.

**Figure 1:**
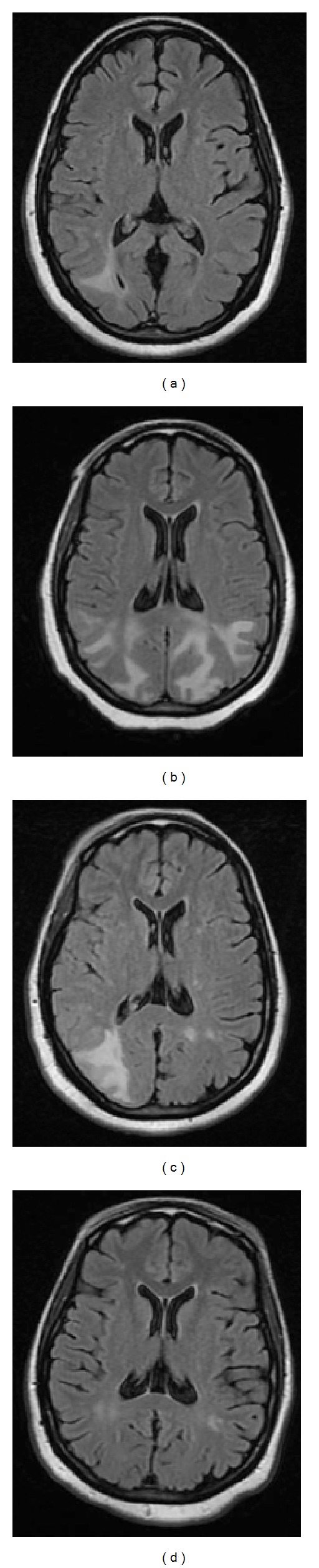
(a) At presentation: axial T2-weighted fluid-attenuated inversion recovery MRI showed multiple large areas of edema in the occipitoparietal regions. (b) At presentation: axial T2-weighted FLAIR MRI showed multiple large areas of edema in the occipitoparietal lobe, with the right greater than the left with no mass effect. (c) At 2-week followup: axial T2-weighted FLAIR MRI showed resolving edema. (d) At 5-month followup: axial T2-weighted FLAIR MRI showed no further edema.

On hospital day 2 the patient was afebrile, nontachycardic, but noted to be hypertensive to 200/100 mmHg. She subsequently experienced two tonic-clonic seizures with right gaze deviation. Laboratory results are shown in Table 1. Emergent brain CT and follow-up brain MRI demonstrated multiple large areas of edema in the occipitoparietal regions on T2 FLAIR images consistent with PRES (Figure 1). Diffusion weighted images showed no restriction.

The patient was treated with lorazepam and phenytoin, and her blood pressure was controlled with metoprolol. She had no further seizures for the remainder of the hospitalization. Her neurologic status returned to her baseline over several days and she was discharged home.

The patient presented again four months later with nausea, vomiting, and headache. The patient was non-adherent with medications and her last dialysis session was 72 hours prior to admission. Upon arrival the patient was afebrile, nontachycardic, with a blood pressure of 215/132 mmHg. The general examination showed the patient was somnolent and partially responsive to verbal and physical stimulation. The remainder of her neurologic exam was normal. She had no visual changes or papilledema. She then experienced a witnessed tonic-clonic seizure in the emergency room. Laboratory results are shown in Table 1.

An ECG was normal. MR images showed multiple large areas of edema in the parieto-occipital lobe, with the right greater than the left with no mass effect (Figure 1). Diffusion weighted imaging showed no evidence of restriction. The patient was treated with labetalol, lorazepam, and phenytoin, and two days later the neurologic findings returned to normal. Follow-up MRI at 2 weeks and 5 months (Figure 1) showed resolution of the edema.

## 2. Discussion

PRES is clinicoradiological syndrome. It is characterized by clinical signs and symptoms of headache, altered mental status, cortical blindness, seizures, and other focal neurological signs [1]. Imaging findings commonly demonstrate edema of the white matter in the posterior portions of the cerebral hemispheres, especially involving the parietooccipital cortex [2, 3]. PRES is distinct from bilateral infarction of posterior cerebral artery area as it rarely involves the calcarine and paramedian occipital-lobe structures [4]. Normal or decreased signal intensity on diffusion sequences and high signal intensity in apparent diffusion coefficient (ADC) maps suggest vasogenic edema rather than cytotoxic edema, again differentiating PRES from acute cerebral infarct. Atypical imaging features are also commonly described, including involvement of the anterior brain or brainstem and the coexistence of ischemia or hemorrhage [5]. 

Categorized under the umbrella of hypertensive encephalopathy, PRES is defined by reversible cerebral edema due to dysfunction of the cerebrovascular blood-brain barrier unit. The pathophysiology of PRES is thought to result from abnormalities in the transmembrane flow of intravascular fluid and proteins caused by two phenomena: (1) cerebral autoregulatory failure and (2) loss of integrity of the blood-brain barrier [6]. A sudden rise of blood pressure, which exceeds the autoregulatory capability of the brain, causes regions of vasodilation and vasoconstriction to develop. The endothelial dysfunction causes a breakdown of the blood-brain barrier allowing for focal transudation of fluid and edema formation [1, 4, 7]. This creates hypertensive encephalopathy with vasogenic edema with clinical and imaging manifestations that resolve with blood pressure control [1]. A range of precipitating etiologies has been described in literature including hypertension, preeclampsia/eclampsia, hypercalcemia, uremia, porphyria, and neurotoxicity secondary to immunosuppressants such as cyclosporine [1, 3, 8].

As in our patient, HIV, too, contributes to endothelial dysfunction. The HIV-1 virus induces activation of the signal transducers and activators of transcription, STAT1 and STAT3, pathway. STAT plays an active role in the cytokine-mediated inflammatory pathways and activates the human brain microvascular endothelial cells, a major component of the blood-brain barrier resulting in inflammation and resulting molecular dysregulation of barrier function [9, 10].

This allows for microbial organisms to enter into the CSF causing the high prevalence of HIV-associated neurocognitive disorders [11]. Also, HIV associated endothelial damage can be seen elsewhere in the body. The imbalance between endothelial progenitors, cell mobilization, and endothelial apoptosis affects the endothelial cell turnover resulting in increased cardiovascular events in HIV patients [12].

There have been three previously reported cases of PRES in HIV patients [4, 13, 14]. The patients were predominantly young adult men aged 33–40 years old presenting with headache and nausea with two of the three patients developing seizures. Two patients presented with hypertension and one presented with hypercalcemia. All patients report CD4 cell counts of 56–126 cells/*μ*L with neuroimaging demonstrating multiple white matter densities involving the periventricular areas. All patients showed clinical and radiological improvements after correction of the underlying cause.

Recurrence of PRES due to various etiologies, though uncommon, does occur. PRES occurrence, as in our patient, is frequently associated with a rapid rise in blood pressure and affects patients that may be predisposed to this cerebral dysregulation. Ergun et al. report a patient on dialysis secondary to end stage renal disease with recurrent PRES [15]. Brain injury may result from sudden pressure changes due to hemodialysis in hypertensive patients with volume overload [16]. However, despite recurrence of shifts in blood pressure and other predisposing factors, recurrence rates are low, ranging from 3.8% to 8% [17, 18]. More data is needed to elucidate the commonalities that lead to recurrence in selected populations.

In this paper, we presented the first case demonstrating recurrent PRES in a HIV patient with end stage renal disease on hemodialysis. The patient's medication nonadherence and volume overload due to intermittent hemodialysis caused uncontrolled hypertension in a patient with likely endothelial cell dysfunction due to HIV and volume shifts during hemodialysis, resulting in PRES. This paper highlights the multifactorial nature of the development of PRES and the need to assess the increased risk of recurrence in patients with multiple risk factors.

## Figures and Tables

**Table 1:** 

Laboratory data	Initial presentation	4 months later
(Variable)	(Value)	(Value)
CSF		
WBC	0	0
RBC	0	0
Glucose	40 mg/dL	59 mg/dL
Protein	28 mg/dL	92 mg/dL
Bacterial culture	Negative	Negative
Fungal culture	Negative	Negative
AFB culture	Negative	
HSV PCR	Negative	
VDRL	Negative	
Cryptococcal Ag	Negative	
WBC	4,000 cells/microL	2,000 cells/microL
Neutrophils	58%	38%
Lymphocytes	28%	54%
Sodium	141 mEq/L	146 mEq/L
Potassium	3.2 mEq/L	5.2 mEq/L
Chloride	102 mEq/L	111 mEq/L
Bicarbonate	25 mEq/L	17 mEq/L
BUN	11 mg/dL	32 mg/dL
Creatinine	5.7 g/dL	12.1 g/dL
